# Ketone Bodies Improve Human CD8^+^ Cytotoxic T-Cell Immune Response During COVID-19 Infection

**DOI:** 10.3389/fmed.2022.923502

**Published:** 2022-06-16

**Authors:** Simon Hirschberger, Luca Gellert, David Effinger, Maximilian Muenchhoff, Markus Herrmann, Josef-Maria Briegel, Bernhard Zwißler, Simone Kreth

**Affiliations:** ^1^Research Unit Molecular Medicine, Department of Anaesthesiology, LMU University Hospital, Ludwig-Maximilian-University Munich (LMU), Munich, Germany; ^2^Walter Brendel Center of Experimental Medicine, Ludwig-Maximilian-University Munich (LMU), Munich, Germany; ^3^Faculty of Medicine, National Reference Center for Retroviruses, Max von Pettenkofer Institute and Gene Center, Virology, Ludwig-Maximilian-University Munich (LMU), Munich, Germany; ^4^COVID-19 Registry of the LMU Munich (CORKUM), LMU University Hospital, LMU Munich, Munich, Germany; ^5^Department of Medicine III, LMU University Hospital, Ludwig-Maximilian-University Munich (LMU), Munich, Germany

**Keywords:** Ketogenic Diet (KD), SARS-CoV-2, COVID-19, T-cell immunometabolism, cytotoxic T cell, metabolic therapy, nutritional immunology

## Abstract

Severe COVID-19 is characterized by profound CD8^+^ T-cell dysfunction, which cannot be specifically treated to date. We here investigate whether metabolic CD8^+^ T-cell reprogramming by ketone bodies could be a promising strategy to overcome the immunoparalysis in COVID-19 patients. This approach was triggered by our recent pioneering study, which has provided evidence that CD8^+^ T-cell capacity in healthy subjects could be significantly empowered by a Ketogenic Diet. These improvements were achieved by immunometabolic rewiring toward oxidative phosphorylation. We here report similar strengthening of CD8^+^ T cells obtained from severely diseased COVID-19 patients: Flow cytometry and ELISA revealed elevated cytokine expression and secretion (up to + 24%) upon ketone treatment and enhanced cell lysis capacity (+ 21%). Metabolic analyses using Seahorse technology revealed upregulated mitochondrial respiratory chain activity (+ 25%), enabling both superior energy supply (+ 44%) and higher mitochondrial reactive oxygen species signaling. These beneficial effects of ketones might represent evolutionary conserved mechanisms to strengthen human immunity. Our findings pave the road for metabolic treatment studies in COVID-19.

## Introduction

Severe acute respiratory syndrome coronavirus 2 (SARS-CoV-2) is keeping the world in suspense for almost 2 years. Still, disease burden remains high and countless patients require hospitalization up to intensive care. Pronounced T-cell immune dysfunction, particularly affecting CD8^+^ T cells, is a hallmark of severe Coronavirus disease 2019 (COVID-19) (1–3). Importantly, an intact cytotoxic T-cell response has been shown to be essential for effective protection against severe COVID-19, control of viral replication and formation of long-lasting immunological memory (4). T-cell function fundamentally depends on cellular metabolism and can be shaped by nutrient availability (5). Consequently, T-cell immune dysfunctions are known to be linked to metabolic alterations (6). Reprogramming CD8^+^ T-cell metabolism could hence be an innovative therapeutic approach to treat the immune malfunction of COVID-19 patients.

In a pioneering study, we have recently provided evidence that a Ketogenic Diet (KD) markedly enhances human T-cell immunity in healthy subjects. On a KD, very limited carbohydrate uptake results in endogenous production of ketone bodies—mainly beta-D-hydroxybutyrate (BHB)—representing evolutionary conserved metabolites, utilized for energy production *via* mitochondrial oxidative phosphorylation (7). We demonstrated that BHB boosters CD8^+^ T-cell function by inducing significantly higher expression of central immune cytokines and enhanced cytotoxicity. These changes were based on a substantial rewiring of T-cell immunometabolism toward mitochondrial oxidative energy production (8).

We thus assumed that ketone bodies might also augment the impaired cytotoxic CD8^+^ T-cell response in severely diseased COVID-19 patients. To test this tempting hypothesis, we analyzed lymphocytes derived from patients with severe SARS-CoV2 infection, consecutively enrolled within the COVID-19 Registry of the LMU Munich (CORKUM) network. In case of favorable immunological results, nutritional intervention could gain substantial importance in the treatment of COVID-19 patients.

## Materials and Methods

### Patient Sampling

Patients are part of the COVID-19 Registry of the LMU University Hospital Munich (CORKUM, WHO trial id DRKS00021225). Patient data were anonymized for analysis and the study was approved by the local ethics committee (No: 20-245 and No. 22-0128). Informed consent was obtained from all patients. Research was performed according to the Declaration of Helsinki (ethical principles for medical research involving human subjects). Immune cells derived from patients with PCR-verified COVID-19 infection, respiratory failure requiring oxygen insufflation and disease severity WHO 4 or above have been included into the experimental immunometabolic intervention. Patient characteristics are depicted in Table 1.

**TABLE 1:** Patient characteristics.

	n	Patient value	Standard value
SOFA score	14	1.4 ± 0.44	
Gender (% female/male)	20	30/70	
Age (years)	20	66.4 ± 19.7	
Oxygen saturation (%)	18	92 ± 2	
Body temperature (°C)	15	37.4 ± 0.2	
O2 Flow (l/min)	18	4.3 ± 0.8	
WHO score	20	4.25 ± 0.14	
Lymphocytes abs (cells/μl)	9	909.1 ± 231.2	1,220–3,560
Lymphocytes rel (%)	9	15.44 ± 4.9	18–46
T cells abs (cells/μl)	9	506.0 ± 98.9	700–2,100
T cells rel (%)	9	63.6 ± 7.3	57–85
Cytotoxic T cells abs (cells/μl)	9	182.4 ± 43.3	200–900
Cytotoxic T cells rel (%)	9	21.0 ± 4.1	10–39
CRP (mg/dl)	20	6.7 ± 1.6	<0.5
IL6 (pg/ml)	20	73.7 ± 16.8	<5.9
PCT (ng/ml)	20	0.2 ± 0.04	<0.1
SARS-CoV2 copy number (copies/ml)	17	116,803,162 ± 97,958,804	<100,000

*All data reported as mean ± SEM.*

*SOFA, Sepsis-related organ failure assessment score; O_2_, oxygen; WHO, World Health Organization; abs, absolute; rel, relative; CRP, c-reactive protein; IL6, interleukin 6; PCT, procalcitonin.*

### Peripheral Blood Mononuclear Cell Culture and Stimulation

Peripheral blood mononuclear cells (PBMC) from patients with verified COVID-19 infection were purified by density centrifugation (Histopaque 1077, Sigma-Aldrich, St. Louis, MO, United States). A ViCell analyzer (Beckman Coulter, Fullerton, CA, United States) was used to determine cell count and viability. Cultivation of PBMC was performed in RPMI 1640 (Invitrogen, Carlsbad, CA, United States) at a glucose concentration of 80 mg/dl. Cell medium was supplemented with 10% heat-inactivated fetal calf serum (Biochrom, Berlin, Germany), 1% L-glutamine (Life Technologies, Carlsbad, CA, United States) and 1% HEPES (Sigma-Aldrich, St. Louis, MO). 100 U/ml penicillin and 100 U/ml streptomycin (Biochrom, Berlin, Germany) were added to prevent bacterial contamination. For incubation with BHB, D/L-beta-hydroxybutyrate (Sigma Aldrich, St. Louis, MO, United States) was added to the medium achieving a final concentration of 10 mM. T-cell stimulation was performed using CD3/CD28 Dynabeads (Thermo Fisher Scientific, Waltham, MA, United States), providing a bead-to-cell ratio of 1:8, and 50 U/ml IL2 (Miltenyi Biotec, Bergisch-Gladbach, Germany). Cells were incubated at 37°C and 5% CO2 for 5 days.

### CD8^+^ T-Cell Cell Isolation

Cytotoxic CD8^+^ T cells were isolated from stimulated PBMC *via* microbead-based separation using the AutoMACSPro Separator following the manufacturer’s instructions (Human CD8 MicroBeads, # 130-045-201, Miltenyi Biotec, Bergisch Gladbach, Germany). Prior to separation, CD3/CD28 Dynabeads were removed magnetically.

### Cytotoxicity Assay

Analysis of CD8^+^ T-cell lysis capacity was carried out using a calcein-acetoxymethyl (AM) lysis assay, cultivating calcein AM-labeled K562 lymphoblasts (target cells) with CD8^+^ T cells (8μM calcein AM; #C1359, Sigma Aldrich, Darmstadt, Germany). Upon cell lysis of target cells, calcein fluorescence was determined on the FilterMax F3 MultiMode Microplate Reader (excitation filter: 480 nm; emission filter: 520 nm | Molecular devices. LLC, San Jose, CA, United States). Relative cell lysis capacity was calculated using the formula [(test release – spontaneous release)/(maximum release – spontaneous release)] × 100.

### Enzyme-Linked Immunosorbent Assay

Quantification of secreted proteins was performed by Enzyme-linked Immunosorbent Assay (ELISA) (IFNγ: #430104; TNFα: #430204; Granzyme B: #439207; Biolegend, San Diego, CA, United States | Perforin: #3465-1HP-2, Mabtech, Nacka Strand, Sweden). Assays were conducted following the manufacturer‘s protocol. Absorbance was measured using a Filtermax F3 and concentrations of target proteins quantified by plate-specific standard curves.

### Oxygen Consumption Rate and Extracellular Acidification Rate

Mitochondrial respiratory and glycolytic capacity were evaluated using a Seahorse XF HS Mini for extracellular flux analysis (Agilent, Santa Clara, United States). CD8^+^ T cells were seeded into the wells of a poly-L-lysine (Biochrom, # L7240, Berlin, Germany) coated 8-well HS mini plate (#103723-100, Agilent, Santa Clara, United States). Seahorse RPMI (#103576-100, Agilent, Santa Clara, United States) supplemented with 1 mM sodium pyruvate, 2 mM glutamine and 5.5 mM glucose served as assay medium. Experiments were run in duplicates or triplicates with 70,000 cells per well. Extracellular acidification rate (ECAR) and oxygen consumption rate (OCR) were measured in response to Mito Stress Test (#103015-100). To this end, final well concentrations of 1 μM Oligomycin, 1 μM FCCP and 0.5 μM Rotenone/Antimycin A were loaded into the respective compound delivery ports of the sensor cartridge and added sequentially during analysis.

### Mitochondrial Membrane Potential

To examine mitochondrial membrane potential ΔψM, the cationic carbocyanine membrane-permeable dye JC1 was used according to the manufacturer’s protocol (Item No. 701560, Cayman Chemical, Ann Arbor, MI, United States). Data were acquired on a FACS Canto II flow cytometer (BD Biosciences, Franklin Lakes, NJ, United States). In intact mitochondria, ΔψM-dependent JC-1 accumulation leads to the formation of J-aggregates that emit red fluorescence (∼ 590 nm). Depolarization of mitochondrial membrane potential results in lower cellular concentrations of the dye, then forming green fluorescent monomeric forms of JC1 (∼ 529 nm). ΔψM is represented as the ratio of the mean fluorescence intensities of red to green. The decoupling agent FCCP (Carbonyl cyanide-p-trifluoromethoxyphenylhydrazone) was applied as a negative control, causing an almost complete disruption of ΔψM.

### Cellular Reactive Oxygen Species

Quantification of intracellular reactive oxygen species (ROS) was performed using CellROX Green (C10492, Thermo Fisher Scientific, Waltham, MA, United States) in accordance with the manufacturer’s instructions. N-acetylcysteine (NAC) and tert-butyl hydroperoxide (TBHP) were used as negative and positive controls, respectively. Analysis was performed on a FACS Canto II flow cytometer (BD Biosciences, Franklin Lakes, NJ, United States).

### Antioxidant Capacity

Cellular antioxidant capacity was evaluated *via* quantification of intracellular glutathione (GSH), using ThiolTracker (T10095, Thermo Fisher Scientific, Waltham, MA, United States) according to the manufacturer’s protocol. Data were acquired on a FACS Canto II flow cytometer (BD Biosciences, Franklin Lakes, NJ, United States).

### Mitochondrial Mass Determination

MitoTracker Green FM (#9074, Cell Signaling Technology, Danvers, MA, United States) was used for flow cytometric determination of mitochondrial mass (200 nM MitoTracker in the dark, 37°C, 15 min). Mitochondrial mass per cell was subsequently obtained by quantification of mean fluorescence intensity (MFI) green on a FACS Canto II flow cytometer (BD Biosciences, Franklin Lakes, NJ, United States).

### Flow Cytometry

Antibody staining for flow cytometric analyses was performed according to the manufacturer’s protocols. First, CD8^+^ T cells were incubated with 2.5 μl Human TruStain FcX™ Fc Receptor Blocking Solution (#422302, BioLegend, San Diego, CA, United States). For extracellular CD4 + /CD8^+^ antibody staining, cells were subsequently incubated on ice with the designated antibody (PerCP anti-human CD8/anti-human CD4, #344707/#317432 BioLegend, San Diego, CA, United States) for a duration of 30 min, protected from light. Intracellular staining of Interferon γ, Granzyme B and Perforin 1 was carried out using FITC anti-human Interferon γ (#502506, BioLegend, San Diego, CA, United States), BV421 anti-human Granzyme B (#396413, BioLegend, San Diego, CA, United States) and APC anti-human Perforin 1 (#308111, BioLegend, San Diego, CA, United States) after cells had been fixed and permeabilized using eBioscience™ Fixation/Permeabilization Concentrate, Diluent and Buffer (#00-5123-43 | #00-5223-56 | #00-8333-56, Invitrogen, Carlsbad, United States) as to the manufacturer’s protocol. Flow cytometry data were acquired on a FACS Canto II (BD Biosciences, Franklin Lakes, NJ, United States). Data analyses were performed using FlowJo v10 (FlowJo, Ashland, United States).

### mRNA Expression Analysis

Expression of mRNA was quantified on a LightCycler 480 instrument (Roche Diagnostics, Mannheim, Germany) as previously described (9, 10). In brief, RNA was isolated using the miRNeasy RNA Isolation Kit (#217004, Quiagen, Hilden, Germany). After on-column DNA digestion, cDNA was synthesized from equal amounts of RNA using a Superscript III reverse transcriptase (Invitrogen, Carlsbad, CA, United States), random hexamers and oligo (dT) primers. Succinate dehydrogenase subunit A (SDHA), and TATA Box Binding Protein (TBP) served as reference genes in all experiments. Primers and probes are specified in Supplementary Table 1. The second derivative maximum method was used to determine quantification cycles by the LightCycler software. Quantification cycle (Cq) cut-off was defined for Cq 35, values beyond cut-off were considered unspecific.

### Statistical Analyses

Statistical analysis was performed using GraphPad Prism 9.2 (GraphPad Software, Inc., United States). Paired *t*-test or Wilcoxon matched-pairs signed rank test, as appropriate, served for comparisons. Normal distribution was tested using the D’Agostino and Pearson test. Data were depicted as mean ± SEM (MFI, OCR and protein data) or as box plots with mean, median, twenty-fifth and seventy-fifth percentiles and range (all other), with dots indicating individual values. *p < 0.05, ^**^p < 0.01. Biological replicates are reported in the figure legends.

## Results

Study subjects exhibited disease severity WHO grade IV or above and respiratory failure requiring continuous oxygen insufflation. Patient characteristics are depicted in Table 1. Lymphocytes were subjected to an established cell culture model (8) and cultivated with D/L-BHB (BHB^+^) under T-cell specific stimulation (CD3/CD28 dynabeads, Supplementary Figures 1A–D). These cells will be referred to as BHB^+^ CD8^+^ T cells.

**FIGURE 1:**
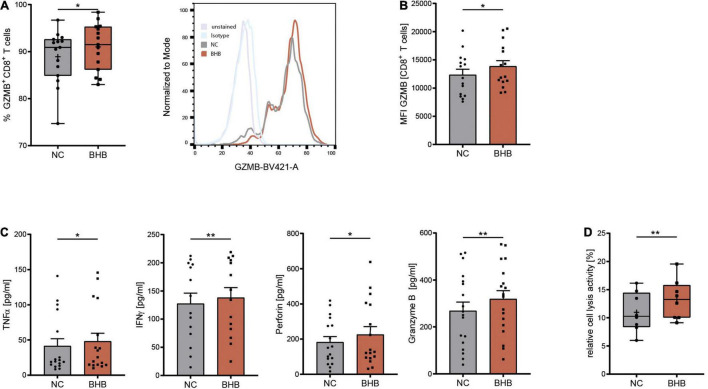
Beta-hydroxybutyrate enhances human T-cell immune capacity during COVID-19. Human peripheral blood mononuclear cells (PBMC) were cultivated for 5 days in RPMI containing 80 mg/dl glucose (NC) and supplemented with 10 mM D/L-beta-hydroxybutyrate (BHB). T-cell stimulation was performed through CD3/CD28 Dynabeads at a bead:cell ratio of 1:8. **(A)** Flow cytometric quantification of CD8^+^ T cells expressing intracellular Granzyme B (left), *n* = 15 individual patients. For better visualization, a histogram example of one patient is shown. **(B)** Mean fluorescence intensity (MFI) Granzyme B per cell, measured in CD8^+^ T cells (right), *n* = 15 individual patients. **(C)** Protein expression of TNFα/IFNγ/Perforin/Granzyme B in the supernatant of stimulated PBMC, *n* = 17/14/17/18 individual patients. **(D)** Relative CD8^+^ cell lysis activity as measured by calcein-fluorescence of isolated CD8^+^ T cells, *n* = 8 individual patients. Paired *t*-test or Wilcoxon matched-pairs signed rank test, as appropriate. *p < 0.05, **p < 0.01.

COVID-19 has been shown to severely impair CD8^+^ T-cell immunity. One hallmark of this cell exhaustion is a decline in cell number (1). Laboratory results of our study cohort corroborate these findings by displaying reduced T lymphocyte and cytotoxic T lymphocyte numbers in COVID-19 patients (Table 1). To evaluate the effect of BHB on the immune function of CD8^+^ lymphocytes, we performed flow cytometric analysis of intracellular cytokines. We detected a significant increase of BHB^+^CD8^+^ T cells expressing the central cytolytic protein granzyme B (Figure 1A) and a substantially augmented granzyme B expression per cell (+ 12% ± 5.4%, *p* = 0.038) (Figure 1B). Functional investigations revealed a profound enhancement of CD8^+^ immune capacity during COVID-19 upon BHB incubation. Secretion of CD8^+^ T-cell cytokines IFNγ (+ 8.3% ± 2.2%, *p* = 0.0026), TNFα (+ 16% ± 7.9%, *p* = 0.0248), Perforin (+ 24% ± 11%, *p* = 0.045) and Granzyme B (+ 19% ± 5.2%, *p* = 0.0023) was markedly elevated (Figure 1C). Consequently, BHB^+^CD8^+^ T cells were found to have significantly increased cell lysis capacity (+ 21% ± 4.9%, *p* = 0.0043; Figure 1D). Taken together, we provide evidence for substantial augmentation of COVID-19 patients’ CD8^+^ T-cell response after treatment with BHB.

**FIGURE 2:**
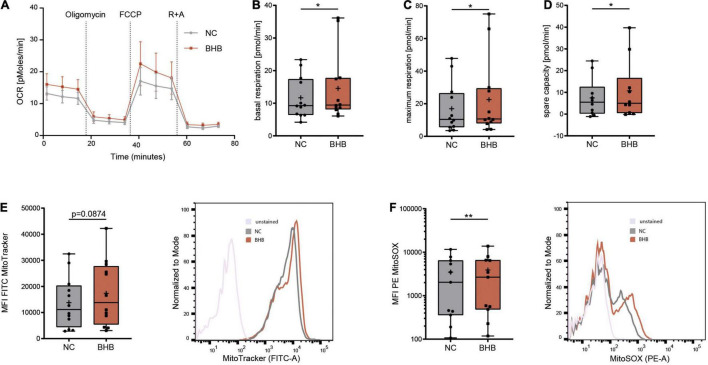
Beta-hydroxybutyrate shifts human T-cell metabolism toward oxidative phosphorylation enabling higher mROS production. Human peripheral blood mononuclear cells (PBMC) were cultivated for 5 days in RPMI containing 80 mg/dl glucose (NC) and supplemented with 10 mM D/L-beta-hydroxybutyrate (BHB). T-cell stimulation was performed through CD3/CD28 Dynabeads at a bead:cell ratio of 1:8. CD8^+^ T cells were isolated *via* magnetic cell separation. **(A–D)** Oxygen consumption rate [OCR] **(A)**, basal **(B)**, maximum **(C)** and spare respiratory capacity **(D)** were measured using a Seahorse HS mini Analyzer, *n* = 5 individual patient samples, each performed in 2–3 technical replicates. **(E)** Mitochondrial mass determined *via* MitoTracker green, indicated by MFI FITC in human CD8^+^ T cells, *n* = 12 individual patient samples. Histogram depicting exemplary change of MitoTracker green. **(F)** Quantification of mitochondrial superoxide production using MitoSOX, displayed as MFI PE in human CD8^+^ T cells, *n* = 11 individual patient samples. Histogram depicting exemplary change of MitoSOX fluorescence. *p < 0.05, **p < 0.01.

In healthy subjects undergoing KD, immunometabolic reprogramming of T cells occurs, which enables higher mitochondrial energy production through oxidative phosphorylation (8). To evaluate whether ketone bodies also enhance CD8^+^ T-cell energy levels during COVID-19, we performed *Seahorse* analyses. BHB^+^CD8^+^ T cells displayed significantly higher basal (+ 17% ± 9.4%, *p* = 0.0463) and maximum respiratory chain activity (+ 34.6% ± 25.3%, *p* = 0.0294) as well as superior spare respiratory capacity (+ 42% ± 30.5%, *p* = 0.0315), providing evidence for strengthened mitochondrial energy production (Figures 2A–D and Supplementary Figure 1D). Of note, this metabolic rewiring did not come at the expense of cellular glycolysis, as extracellular acidification rate did not decrease in BHB^+^CD8^+^ T cells (Supplementary Figure 1E). These functional results were complemented by structural analyses, showing a trend toward increased mitochondrial mass in BHB^+^CD8^+^ T cells (+ 26% ± 17.7%; Figure 2E). Collectively, these data indicate that ketone bodies direct human CD8^+^ T cells toward aerobic mitochondrial metabolism during COVID-19, thereby enabling superior energy supply.

Mitochondrial respiratory chain complexes are the main source for reactive oxygen species (ROS) (11). Mitochondrial [m]ROS are indispensable for an adequate T-cell immune response (12, 13). Thus, we investigated whether augmented ROS production due to elevated OXPHOS activity in BHB^+^CD8^+^ T cells provides an additional basis for the reported augmentation of cytotoxic immune function during COVID-19. Indeed, mitochondrial ROS were significantly elevated after incubation with ketone bodies (+ 12% ± 5.9%, *p* = 0.0029, Figure 2F). Of note, we did not detect uncontrolled expansion of ROS, as cellular levels of ROS remained unchanged (Supplementary Figure 2A), highlighting the concept of mitohormesis (14, 15). Consequently, neither cellular expression of anti-oxidative glutathione nor the integrity of the mitochondrial membrane were impaired upon incubation with ketone bodies (Supplementary Figures 2B,C). Of note, analysis of CD4^+^ T-cells showed no alteration to mitochondrial mass or ROS production in response to BHB (Supplementary Figures 2D–H). In conclusion, these findings demonstrate an increase of [m]ROS production serving as T-cell second messenger in BHB^+^CD8^+^ T cells without compromising cell viability.

## Discussion

We have recently reported a strong positive impact of a KD on human T-cell immune capacity in healthy volunteers (8). In the current study, we found the same pattern of effects in disease: The attenuated CD8^+^ T-cell functions of severely diseased COVID-19 patients were significantly empowered. Again, this phenomenon was based on a higher respiratory capacity -enabling superior energy production- and increased mitochondrial ROS which serve as T-cell second messenger. It is conceivable that the observed effects of ketone bodies represent evolutionary conserved mechanisms for stabilizing human immunity in health and disease.

Functional exhaustion of T-cells is known to be linked to mitochondrial dysfunction. Thus, we hypothesized that mitochondrial empowerment through BHB could improve T-cell function during COVID-19. We provide evidence for an increased energetic capacity of BHB^+^CD8^+^ T-cells during COVID-19. T cells are capable of using BHB *via* Krebs cycle oxidation, which is known to fuel OXPHOS with superior efficacy (16, 17). Therefore, augmented cellular energy supply through metabolization of BHB could enable an enhanced immune response of CD8^+^ T-cells.

Elevated mitochondrial oxidative phosphorylation translates into increased mROS, as respiratory chain complexes are the major source of mROS (18). T-cell activation and function inevitably relies on mROS, rendering them a pivotal signaling molecule for T-cell immunity (12, 19). We show increased mROS in response to BHB, which might provide the second immunometabolic basis for the augmented CD8^+^ T-cell immune capacity.

Patients PBMC were incubated using 10 mM D/L-BHB. As only D-BHB is metabolically active (7, 20–22), this refers to 5 mM D-BHB, which is similar to maximum blood ketone levels achievable *via ad libitum* KD (8). Since *in vitro* no BHB synthesis is occurring, higher initial BHB concentrations must be used to ensure adequate ketosis during cell culture. Of note, BHB concentration in cell culture medium at the end of incubation was in the range of 2 mM, thus almost identical to mean blood BHB *in vivo* (Supplementary Figure 2I). Accordingly, lower initial concentrations of BHB *in vitro* did not evoke comparably positive effects on human T-cell immunometabolism. On a KD, exogenous supply of ketone esters or the use of MCT oil could help to achieve blood ketone levels close to 2 mM BHB.

In our previous investigations, KD had only a limited effect on CD4^+^ T-cells. In the current study, again, CD4^+^ T-cells do not respond to BHB. CD4^+^ T cells are essential regulators of the human immune system (23). They can be further divided into distinct subsets with individual immunological function and metabolic characteristics (24, 25). We assume that this subset heterogeneity might be responsible for amelioration of the overall effect of KD when analyzing bulk CD4^+^ T-cells. Thus, further studies are required to dissect the impact of KD on individual CD4^+^ T-cell subsets in health and disease.

Due to their multidimensional beneficial immunometabolic effects, ketone bodies have been proposed as a countermeasure against viral infections (26). To date, no study investigated the impact of BHB on human viral infections. In mice, BHB has already shown to induce a protective immune response against influenza virus infection (27). Similarly, in beta coronavirus-infected mice, KD improved γδ T-cell immunity and dampened inflammation (28). The potential benefit of KD also expands to other immune cell types. Macrophages contribute to detrimental immune responses to COVID-19, which could be ameliorated through redirection of M1 to M2 phenotype *via* metabolic rewiring on a KD (29). Beyond immunological effects, KD has been proposed as a metabolic therapy against COVID-19 through restoration of systemic energy metabolism (30). Of note, KD *in vivo* might evoke additional positive effects: carbohydrate restriction results in diminished levels of glucose and insulin, thus breaking the cycle of glucose-insulin-dependent inflammation and immunosuppression (31–36). This could be of exceptional importance, since metabolic comorbidities have a devastating impact on COVID-19 patients (37–40).

Consequently, a retrospective data analysis of COVID-19 patients on a KD revealed correlations to a reduced mortality (41). Prospective randomized clinical trials will now have to evaluate the precise impact of BHB on human metabolism *in vivo* during COVID-19.

Our study paves the road for the development of metabolic treatment strategies against COVID-19, which now have to be evaluated within the framework of controlled prospective studies. Of note, the required nutrition formula and protocols are already available. A clinical trial evaluating the impact of KD in sepsis patients has already finished the enrolling phase, and results are expected in the near future (42).

## Data Availability Statement

The original contributions presented in the study are included in the article/Supplementary Material, further inquiries can be directed to the corresponding author/s.

## Ethics Statement

The studies involving human participants were reviewed and approved by LMU Ethics Committee. The patients/participants provided their written informed consent to participate in this study.

## Author Contributions

SH designed the study, planned, supervised and conducted the experiments, prepared figures, and wrote the manuscript. LG conducted experiments and participated in writing the manuscript. DE participated in figure preparation and writing of the manuscript. MM, MH, J-MB, and BZ participated in the study design. SK supervised the study and wrote the manuscript. All authors contributed to the interpretation of the data and approved the final version of the manuscript.

## Conflict of Interest

The authors declare that the research was conducted in the absence of any commercial or financial relationships that could be construed as a potential conflict of interest.

## Publisher’s Note

All claims expressed in this article are solely those of the authors and do not necessarily represent those of their affiliated organizations, or those of the publisher, the editors and the reviewers. Any product that may be evaluated in this article, or claim that may be made by its manufacturer, is not guaranteed or endorsed by the publisher.
