# Supplementary material for: Ketone Bodies Improve Human CD8+ Cytotoxic T-Cell Immune Response During COVID-19 Infection
Source: Front Med (Lausanne). 2022 Jun 16;9:923502. doi: 10.3389/fmed.2022.923502 (PMC9243504; doi:10.3389/fmed.2022.923502)
Supplement: Supplementary file 1 [file Data_Sheet_1.PDF]

Supplementary Material

Ketone Bodies improve human $CD8^+$ cytotoxic T-cell immune response during COVID-19 infection

Simon Hirschberger[1,2]; Luca Gellert[2]; David Effinger[1,2]; Maximilian Münchhoff[3,5]; Markus Herrmann[4,5]; Josef-Maria Briegel[1,5]; Bernhard Zwißler[1,5]; Simone Kreth[1,2,*]

1 Department of Anaesthesiology, Research Unit Molecular Medicine, LMU University Hospital, Ludwig-Maximilian-University Munich (LMU), Munich, 81377, Germany.

2 Walter Brendel Center of Experimental Medicine, Ludwig-Maximilian-University Munich (LMU), Munich, 81377, Germany.

3 Max von Pettenkofer Institute and Gene Center, Virology, National Reference Center for Retroviruses, Faculty of Medicine, Ludwig-Maximilian-University Munich (LMU), Munich, 81377, Germany.

4 Department of Medicine III, LMU University Hospital, Ludwig-Maximilian-University Munich (LMU), Munich, 81377, Germany.

5 COVID-19 Registry of the LMU Munich (CORKUM), LMU University Hospital, LMU Munich

*** Correspondence:**
Simone Kreth, MD, PhD. Walter Brendel Center of Experimental Medicine, Ludwig-Maximilian-University Munich (LMU). Marchioninistreet 68, 81377, Munich, Germany. simone.kreth@med.uni-muenchen.de. +49 - 89 - 2180 76508

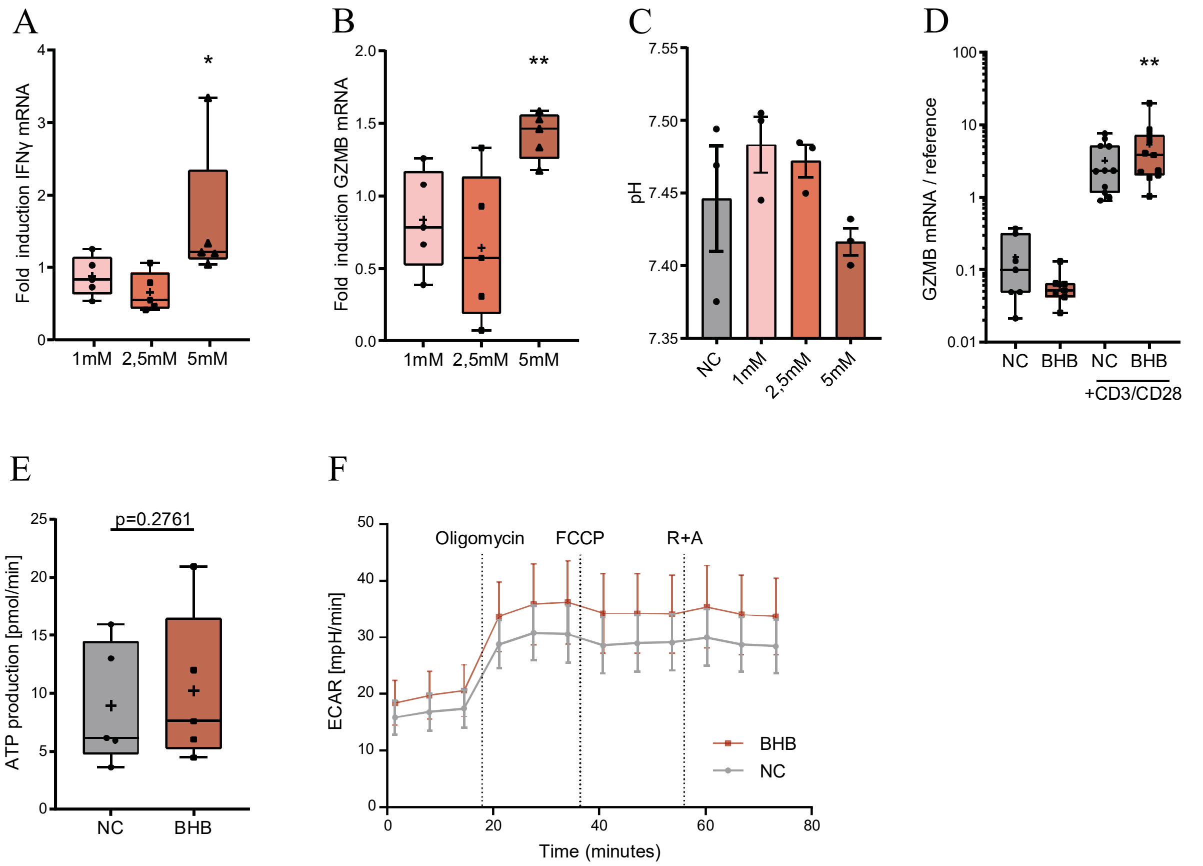

Figure S1 Elevated ATP production and ECAR in response to BHB

Human peripheral blood mononuclear cells (PBMC) were cultivated for 5d in RPMI containing 80mg/dl glucose (NC) and supplemented with 2mM, 5mM and 10mM D/L-beta-hydroxybutyrate (Figure S1A/B) or only 10mM D/L-beta-hydroxybutyrate (BHB | S1C/D). T-cell stimulation was performed through CD3/CD28 Dynabeads at a bead:cell ratio of 1:8. $CD8^+$ T cells were isolated via magnetic cell separation. Fold induction of **A** IFNγ and **B** GZMB mRNA relative to normal control, quantified in human T cells cultivated with varying concentrations of D-BHB (corresponding to 2mM, 5mM and 10mM racemic D/L-BHB) as indicated, n=5 biological replicates. **C** pH of cell culture in response to various BHB concentrations as indicated, measured on a Siemens RAPIDlab 1265, n=3. **D** mRNA expression of GZMB in unstimulated and CD3/CD28-stimulated human T cells relative to internal controls, n=7/7/11/11 biological replicates. **E** ATP production and **F** Extracellular Acidification Rate (ECAR) were measured using a *Seahorse* HS Mini Analyzer, n=5 individual patient samples, each performed in 2-3 technical replicates. Data depicted as mean ± SEM (ECAR) and box plots with median, twenty-fifth and seventy-fifth percentiles and range (all other). Dots indicating individual values. *p<0.05, **p<0.01, paired t-test/ Wilcoxon matched-pairs signed rank test, as appropriate.

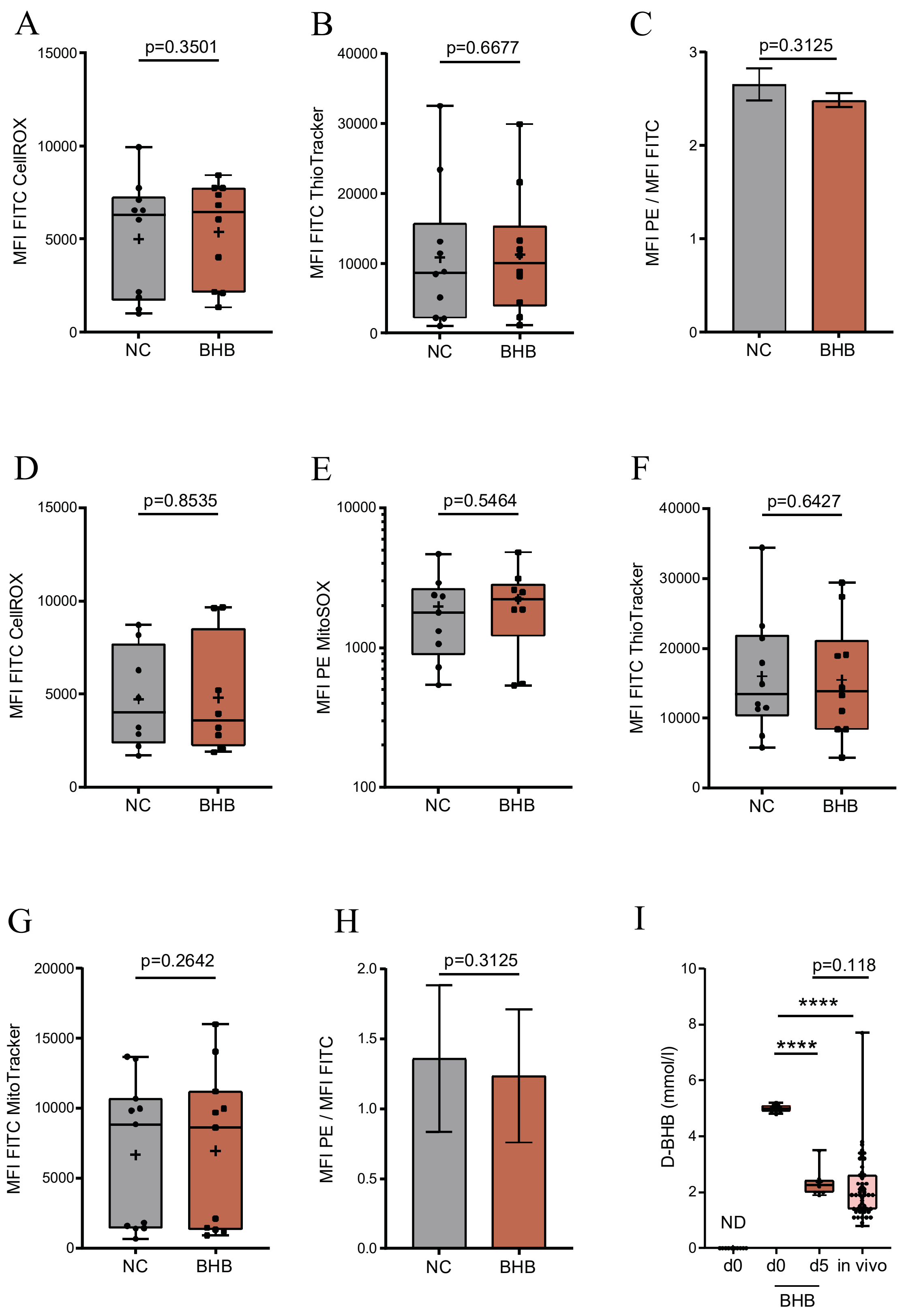

Figure S2 Sustained mitohormesis in response to BHB and no effect of BHB on CD4 cells

Human peripheral blood mononuclear cells (PBMC) were cultivated for 5d in RPMI containing 80 mg/dl glucose (NC) and supplemented with 10mM D/L-beta-hydroxybutyrate (BHB). T-

cell stimulation was performed through CD3/CD28 Dynabeads at a bead:cell ratio of 1:8. $CD8^+$ T cells were isolated via magnetic cell separation. **A** Cellular reactive oxygen species (ROS) quantified through CellROX, indicated by MFI FITC in human $CD8^+$ T cells, n=10 individual patient samples. **B** Analysis of cellular glutathione content using ThioTracker, depicted as MFI FITC in human $CD8^+$ T cells, n=10 individual patient samples. **C** Evaluation of mitochondrial membrane potential using JC-1, analyzed as MFI PE/ MFI FITC in human $CD8^+$ T cells, FCCP served as the negative control, n = 6 individual patient samples. **D** Cellular reactive oxygen species (ROS) quantified through CellROX, indicated by MFI FITC in human $CD4^+$ T cells, n=8 individual patient samples. **E** Quantification of mitochondrial superoxide production using MitoSOX, displayed as MFI PE in human $CD4^+$ T cells, n=9 individual patient samples. **F** Analysis of cellular glutathione content using ThioTracker, depicted as MFI FITC in human $CD4^+$ T cells, n=11 individual patient samples. **G** Mitochondrial mass determined via MitoTracker green, indicated by MFI FITC in human $CD4^+$ T cells, n=10 individual patient samples. **H** Evaluation of mitochondrial membrane potential using JC-1, analyzed as MFI PE/ MFI FITC in human $CD4^+$ T cells, FCCP served as the negative control, n = 5 individual patient samples. **I** Concentration of D-BHB in cell culture supernatant of patient PBMC at the start (d0, NC and BHB) and at the end (d5) of T-cell stimulation, compared to in vivo serum D-BHB concentration of healthy volunteers on a three-weeks KD, measured at the end of the diet. D-BHB quantified by point of care testing using a Glucomen Aero 2K (Berlin Chemie AG, Berlin, Germany), n=10/10/10/49, ND=not detectable. Data depicted as mean ± SEM (JC1) and box plots with median, twenty-fifth and seventy-fifth percentiles and range (all other). Dots indicating individual values. *p<0.05, **p<0.01, ****p<0.0001, paired t-test/ Wilcoxon matched-pairs signed rank test, as appropriate.

Table S1 Primers and Probes

Target	Primer for	Primer rev	Probe
TBP	5’ GAACATCATGGATCAGAACAACA 3’	5’ ATAGGGATTCCGGGAGTCAT 3’	87
SDHA	5’ GAGGCAGGGTTTAATACAGCA 3’	5’ CCAGTTGTCCTCCTCCATGT 3’	132
GZMB	5’ GGGGGACCCAGAGATTAAAA 3’	5’ CCATTGTTTCGTCCATAGGAG 3’	37
IFNy	5’ GGCATTTTGAAGAATTGGAAAG 3’	5’ TTTGGATGCTCTGGTCATCTT 3’	21
